# Resequencing of the *Lactobacillus plantarum* Strain WJL Genome

**DOI:** 10.1128/genomeA.01382-15

**Published:** 2015-11-25

**Authors:** Maria Elena Martino, Jumamurat R. Bayjanov, Pauline Joncour, Sandrine Hughes, Benjamin Gillet, Michiel Kleerebezem, Roland Siezen, Sacha A. F. T. van Hijum, François Leulier

**Affiliations:** a Institut de Génomique Fonctionnelle de Lyon (IGFL), École Normale Supérieure de Lyon, CNRS UMR 5242, Université Claude Bernard Lyon 1, Lyon France; b Center for Molecular and Biomolecular Informatics, Nijmegen, The Netherlands; c Host Microbe Interactomics Group, Wageningen University, Wageningen, The Netherlands; d Microbial Bioinformatics, Ede, The Netherlands; e NIZO food research, Ede, The Netherlands

## Abstract

*Lactobacillus plantarum* strain WJL is a symbiont isolated from the *Drosophila melanogaster* gut. The genome of *L. plantarum* WJL, first sequenced in 2013, was resequenced and rescaffolded in this study. A combination of Sanger and Illumina sequencing allowed us to reduce the number of contigs from 102 to 13. This work contributes to a better understanding of the genome and function of this organism.

## GENOME ANNOUNCEMENT

*Lactobacillus plantarum* is a lactic acid bacterium commonly found in a variety of niches, such as plants, gastrointestinal tracts of human and animals, and foods, like meat, fish, vegetables, and raw and fermented dairy products (1). *L. plantarum* strain WJL was originally isolated from the intestine of the fruit fly, *Drosophila melanogaster*, and is considered a symbiont, providing several benefits to its host (2). A recent study demonstrated that *L. plantarum* WJL is capable of promoting systemic larval growth by controlling hormonal signaling (3). However, the molecular mechanisms through which strain WJL exerts its beneficial influence are still largely undefined.

The initially sequenced genome of *L. plantarum* WJL was published in 2013 (2) and comprises 102 contigs. In the current study, we resequenced its genome using a combination of Illumina and Sanger sequencing. This allowed closing of most of the original gaps, significantly reducing the number of contigs.

The genome sequence of *L. plantarum* WJL was determined using a 150-bp paired-end library with Illumina MiSeq technology (Illumina, San Diego, CA) at ProfileXpert (Lyon, France). A total of 1,711,744 reads were generated and assembled into 38 contigs using Ray (4), ranging from 560 bp to 1,586,832 bp. Gap closing and resequencing of low-quality regions were conducted by Sanger sequencing to arrive at a high-quality finished genome sequence. Thirty-eight gap-closing primer pairs were designed between contigs using the Primer3 software (5). The endpoint PCRs were conducted on a Veriti Applied Biosystems thermocycler (Life Technologies, Carlsbad, CA). Twenty-five gaps were closed, leading to a total of 13 contigs. Functional annotations of the predicted genes were performed using the RAST server (6). The resequencing of *L. plantarum* WJL led to a final draft genome sequence of 3,503,067 bp, which contains 3,477 open reading frames (ORFs), allowing the identification of 67 new protein-coding sequences. Sixteen rRNA genes and 81 tRNA genes were identified. The G+C content of the genome is 44.2%. Interestingly, genome analysis of the resequenced *L. plantarum* WJL revealed structural differences, such as the presence of 12 and 10 additional rRNA and tRNA genes, respectively, compared with the previously published sequence (2).

The genome information of *L. plantarum* WJL presented in this study will be useful for further studies, such as the analysis of its role as symbiont, with the aim of dissecting the molecular basis of gut-microbiota interactions.

### Nucleotide sequence accession number.

This whole-genome shotgun project has been deposited at DDBJ/EMBL/GenBank under the accession no. LKLZ00000000.
